# Comparative clinical and thermographic responses to dexamethasone and saline solution mesotherapy in horses with spinal disorders

**DOI:** 10.3389/fvets.2026.1839834

**Published:** 2026-06-30

**Authors:** Tainã Kuwer Jacobsen, Marcos da Silva Azevedo, Sandro Colla, Anderson Ramos Carvalho, Flavio Desessards De La Corte, Grasiela De Bastiani

**Affiliations:** 1 Graduate Program in Animal Medicine: Equines, Federal University of Rio Grande do Sul, Porto Alegre, Brazil; 2 Department of Large Animal Clinical Sciences, University Veterinary Hospital, Federal University of Santa Maria, Santa Maria, Brazil; 3 Department of Large Animal Clinical Sciences, Equine Performance and Rehabilitation Center, University of Tennessee College of Veterinary Medicine, Knoxville, TN, United States; 4 Laboratory of Chemometrics and Analytical Instrumentation, Institute of Chemistry, Federal University of Rio Grande do Sul, Porto Alegre, Brazil; 5 Department of Animal Medicine, School of Veterinary Medicine, Federal University of Rio Grande do Sul, Porto Alegre, Brazil

**Keywords:** axial dysfunction, cervical spine, dexamethasone, horses, mesotherapy, spinal disorders, thermography, thoracolumbar spine

## Abstract

**Background:**

Back pain and spinal dysfunction in horses are multifactorial conditions frequently associated with musculoskeletal alterations that may impair mobility, performance, and welfare. Mesotherapy has emerged as a minimally invasive adjunctive approach for managing musculoskeletal disorders; however, comparative clinical responses to different intradermal protocols in equine spinal conditions remain insufficiently investigated. The present study aims to compare the clinical and thermographic responses of two mesotherapy protocols in horses diagnosed with cervical or thoracolumbar spinal disorders.

**Methods:**

Forty horses with ultrasonographically confirmed spinal alterations were stratified by anatomical region (cervical or thoracolumbar) and randomly allocated into four subgroups (*n* = 10/group) to receive intradermal mesotherapy with either saline solution or dexamethasone sulfate (0.05 mg/kg). Clinical assessments of joint mobility and muscle hypotrophy, along with thermographic evaluations, were performed at baseline and at 48 h, 15, 30, and 60 days after treatment. Data were analyzed using linear mixed models with horses included as random effects for repeated measures, followed by Dunnett’s *post-hoc* test (*p* < 0.05).

**Results:**

Temporal improvements were observed in joint mobility and thermographic with a progressive improvement in thoracolumbar extension mobility observed from 48 h (*p* < 0.0001) 15 days (*p* = 0.0003), 30 days (*p* = 0.0006), and 60 days (*p* = 0.0027), and significant improvements in thoracolumbar thermographic patterns at 48 h (*p* = 0.0219) and 15 days (*p* < 0.0001). No statistically significant differences were detected between dexamethasone and saline-based mesotherapy protocols for most evaluated outcomes under the experimental conditions tested. Thermographic assessment appeared comparatively more responsive in detecting temporal pattern changes under the semiquantitative study conditions.

**Conclusion:**

Under the present experimental conditions, both mesotherapy protocols were associated with comparable clinical and thermographic responses over time. However, given the absence of untreated or sham-treated control groups, these findings should be interpreted as comparative responses between active protocols rather than definitive evidence of therapeutic efficacy or mechanistic action. Further controlled studies are warranted to clarify therapeutic effects and procedural contributions.

## 1 Introduction

Back pain in horses is a complex condition frequently linked to changes in the musculoskeletal system, including bone changes such as osteoarthritis of the articular process joints, spondylosis, and overriding dorsal spinous processes (1–4). Soft tissue disorders also play a significant role, with notable conditions including muscle hypertonicity, myositis, hypotrophy, desmopathies, and enthesopathies (5). External factors such as trauma, neurological dysfunctions, overload from excessive exercise, improper saddle use, and rider weight can aggravate or trigger the painful condition (6–8).

The most common clinical signs of the equine axial skeleton dysfunction include reduced joint mobility, increased discomfort-related sensitivity during vertebral palpation, vertebral misalignment, muscle asymmetry (due to hypotrophy or compensatory hypertrophy), altered movement biomechanics, and, consequently, decreased athletic performance (9, 10). Equine spinal disorders may be associated with acute or chronic musculoskeletal pain depending on lesion chronicity, tissue involvement, and compensatory biomechanical overload (4). Persistent nociceptive stimulation may contribute to pain sensitization phenomena, including increased mechanical sensitivity (11); however, objective characterization of pain phenotypes was beyond the scope of this study.

With advancements in imaging techniques and functional evaluation of the axial skeleton, new therapeutic approaches have been developed for managing back pain in horses, spinal disorders, and axial dysfunction. Although the use of systemic corticosteroids and non-steroidal anti-inflammatory drugs (NSAIDs) remains a common practice, there is a growing trend toward adopting integrative and minimally invasive therapies to reduce dependence on systemic pharmacological treatments (12). Therapeutic options include physical rehabilitation, chiropractic care, acupuncture, physiotherapy, extracorporeal shockwave therapy, mesotherapy, and ultrasound-guided intraarticular and paravertebral injections, providing more effective and targeted management of equine spinal pathologies (1, 12–14).

Mesotherapy, also known as local intradermal therapy, is a minimally invasive technique that involves the administration of pharmacological substances directly into the dermis using micro-needles (15). First described in 1958 by French physician Michel Pistor, this approach aims to reduce the need for systemic drug doses, thereby minimizing the side effects associated with prolonged medication use. The method allows for the application of reduced quantities of drugs directly to the affected area, promoting a more localized therapeutic effect (16).

Although the mechanism of action of mesotherapy is not fully elucidated, three main theories have been proposed. (a) Local pharmacological effect: this theory suggests that the injected substances act directly on the tissues and cells in the affected area, providing a significant therapeutic effect with minimal systemic action (17, 18). (b) Mechanical stimulation: It is proposed that microneedling promotes vasodilation, increases local blood flow, and stimulates the release of inflammatory mediators, thereby enhancing tissue repair and regenerative processes (19). (c) Neural modulation (gate control theory): this theory proposes that cutaneous stimulation may modulate afferent nociceptive transmission through sensory and spinal inhibitory pathways, potentially contributing to pain modulation (16).

The growing interest in integrative and minimally invasive therapies in both human and veterinary medicine is driven by the adverse effects associated with the chronic use of systemic corticosteroids and the risks inherent to more invasive procedures (12). In this context, mesotherapy has emerged as a promising alternative for managing back pain and musculoskeletal disorders in different species. Retrospective studies have demonstrated its effectiveness in pain control in humans (20–25), dogs (26) and horses (27), highlighting its potential as a complementary therapeutic option for the treatment of both chronic and acute pain in equine athletes.

The present study aims to compare the clinical and thermographic responses of two mesotherapy protocols administered through mesotherapy in horses diagnosed with cervical or thoracolumbar spinal disorders. Additionally, we aim to investigate clinical outcomes through subjective assessment of joint mobility, muscle hypotrophy, and thermographic parameters, with the goal of comparing the relative responsiveness of these variables for detecting temporal clinical changes. We hypothesized that both treatments would yield similar outcomes and that the clinical outcomes would differ in their ability to detect therapeutic responses.

## 2 Materials and methods

### 2.1 Horses and group division

Out of a population of 76 horses, 40 animals (52.63%) were selected. The inclusion criteria comprised horses presenting ultrasonographically identified cervical or thoracolumbar spinal abnormalities (grades I–III), associated with clinical indicators potentially consistent with axial dysfunction, including alterations in joint mobility, muscle asymmetry/hypotrophy, thermographic findings, palpation sensitivity, and locomotor assessment. Historical clinical information from riders and handlers, including discomfort-related reactions during saddling, was also considered during clinical characterization. However, overt pain sensitivity and lameness were not mandatory inclusion criteria, and locomotor abnormalities were interpreted cautiously due to their multifactorial etiology. The horses had a mean age of 15 ± 5.5 years, a mean body mass of 500 ± 50 kg, consisted of 35 males (87.5%) and 5 females (12.5%), mixed breeds and were located at the Mounted Police Station in Southern Brazil. Throughout the study, all horses maintained their regular work and training routines without any rest periods or significant changes in intensity. No *a priori* sample size or formal statistical power calculation was performed. The study population was defined as a convenience sample composed of horses that were available at the study site and met the predefined clinical and ultrasonographic eligibility criteria. The present study was approved by the National Council for Control of Animal Experimentation (CONCEA) and by the Ethics Committee on Animal Use of the Federal University of Santa Catarina (CEUA/UFSC), protocol number CEUA: 6610200721.

### 2.2 Ultrasonographic evaluation of the axial skeleton

The horses underwent ultrasonographic evaluation of the axial skeleton from C1 to C7 and T7 to L6 on both sides. It is noteworthy that cervical or thoracolumbar trichotomy was not permitted and not performed. A solution of 70% alcohol and the conductive gel was used to enhance transducer coaptation and the visualization of the structures. Ultrasonographic images were generated using a Sonoscape SW^®^ (Guangdong, China), device equipped with a convex probe of 3–5 MHz. Transverse and longitudinal ultrasonographic sections were performed, examining articular surfaces, capsules, joint spaces, and bone surfaces (28, 29).

Ultrasonographic images were classified as grade 0—no abnormality; grade I—mild irregularity of articular facets, joint space reduction, and capsulitis; grade II—moderate irregularity of articular facets and joint space reduction, and the presence of osteophytes; and grade III—marked irregularity of articular facets and joint space reduction, and the presence of osteophytes. The ultrasonographic study was conducted as part of the animal inclusion process, thus, images were obtained only at the pre-treatment time point (T0). Horses with cervical spine abnormalities (grade I, II, and III) were selected for Group 1, and horses with thoracolumbar spine abnormalities (grade I, II, and III) were selected for Group 2.

### 2.3 Clinical evaluation of the axial skeleton

**Figure 1:**
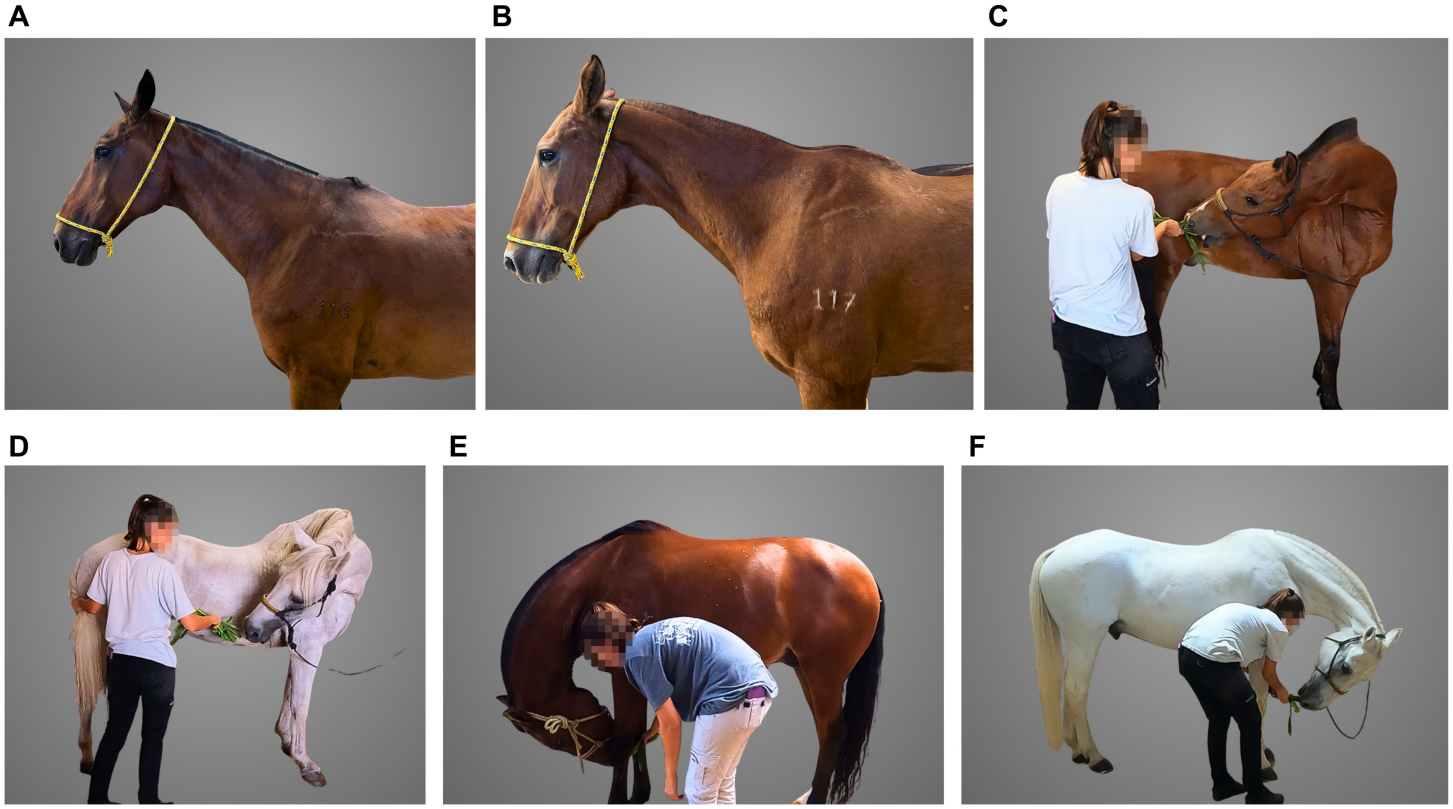
Clinical evaluation of the equine cervical spine. **(A,B)** Static visual inspection of the left cervical region. **(A)** Mild cervical muscle hypotrophy. **(B)** Marked cervical muscle hypotrophy. **(C)** Food stimulus used to induce right cervical lateralization without abnormality. **(D)** Example of reduced cervical lateralization. **(E)** Food stimulus used to induce cervical flexion without abnormality. **(F)** Example of reduced cervical flexion.

Clinical evaluations of the axial skeleton were conducted at the following points: T0, 48 h (T1), 15 days (T2), 30 days (T3), and 60 days after treatment (T4). Visual inspection and palpation of the cervical spine were performed to identify muscle hypertonicity, hypotrophy, pain sensation, and swelling (Figures 1A,B). Pain sensitivity was assessed qualitatively through observable discomfort-related responses during palpation or manipulation, as interpreted by the blinded evaluator. This variable was recorded descriptively and was not designed as a longitudinal outcome measure. A gentle pressure was applied over the atlantoaxial joint (C1-C2), seeking a possible painful reaction. Food (grass or grain) was offered to provoke lateral bending to the right and then to the left, prompting the horse to perform the lateralization movement of the neck, directing the head to the region near the femorotibial patellar joint (Figures 1C,D). The food was also offered between the carpal or front fetlock joints to assess cervical flexion (adapted 30) (Figures 1E,F).

**Figure 2:**
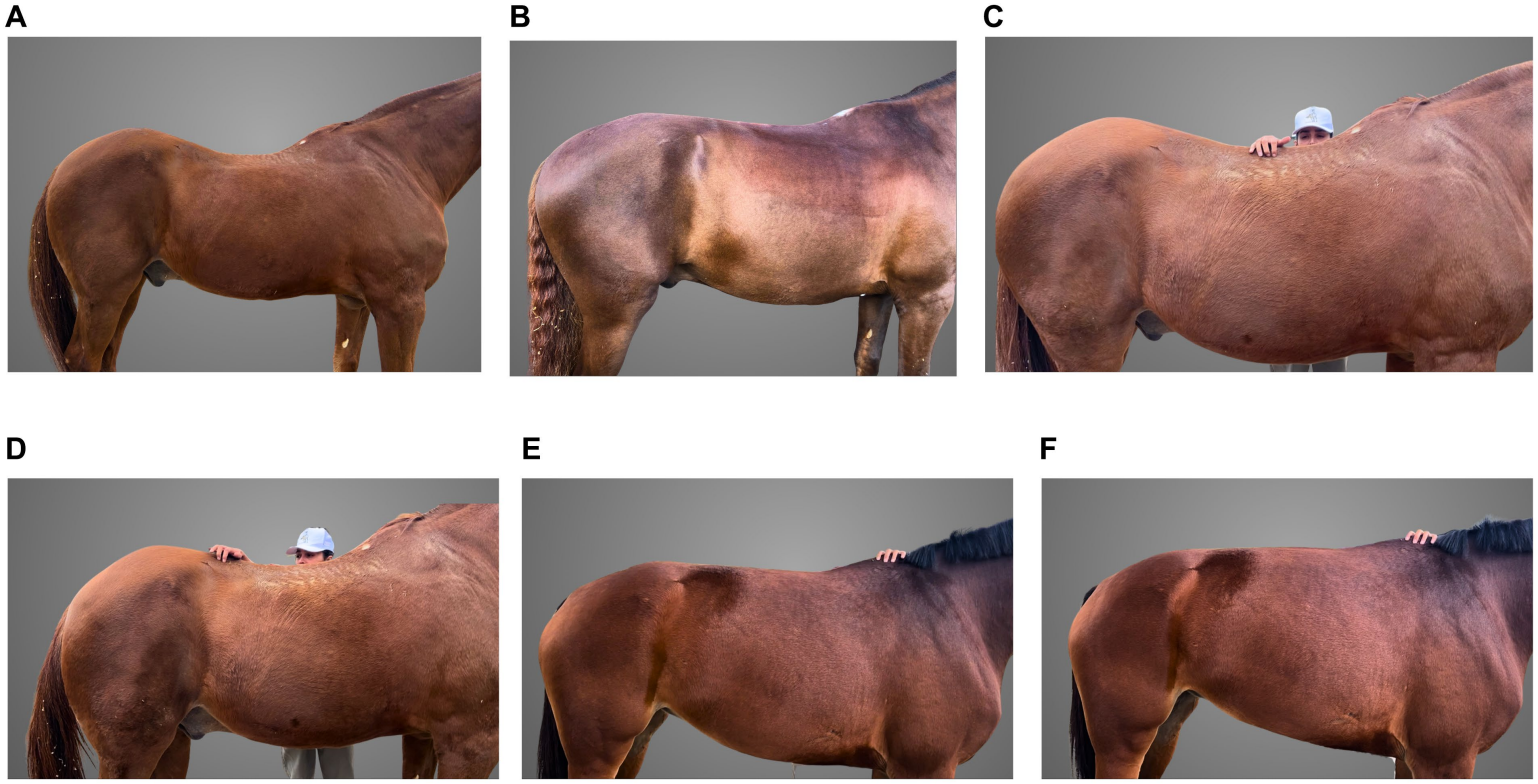
Clinical evaluation of the equine thoracolumbar spine. **(A)** Marked thoracic lordosis identified during static visual inspection. **(B)** Slight thoracic muscle hypotrophy. **(C)** Palpation and pressure test of the thoracic region. **(D)** Palpation and pressure test of the lumbar region. **(E)** Pressure stimulus applied to the ventral abdominal region over the linea alba, cranial to the umbilical scar, to induce thoracolumbar flexion. **(F)** Thoracolumbar flexion without abnormality.

Clinical evaluation of the thoracolumbar spine also began with visually inspecting the entire segment to identify muscle hypertonicity, muscle hypotrophy, focal or regional swelling, and postural alterations such as scoliosis, lordosis, or kyphosis (Figures 2A,B). Subsequently, the entire spine length was palpated, and joint mobility and pain sensitivity were assessed on palpation. The digital pressure was applied along the whole length of the thoracolumbar spine (longissimus dorsi), where a healthy horse is expected to demonstrate spinal extension without marked discomfort-related responses (Figures 2C,D). Next, a gentle touch was applied to the ventral region of the abdomen, over the linea alba cranially to the umbilical scar, inducing the flexion movement of the thoracolumbar spine (adapted 30) (Figures 2E,F).

The clinical assessment of cervical and thoracolumbar mobility was performed using investigator-developed semiquantitative ordinal scoring systems created for longitudinal comparative evaluation in this study. Score classification was informed by previously described clinical examination methods for spinal mobility assessment (30). Joint mobility was classified as score 0—no abnormalities; score 1—slight reduction; score 2—mild reduction; score 3—moderate reduction; and score 4—marked reduction in mobility. Muscle hypotrophy was assessed using an investigator-developed semiquantitative visual scoring system informed by previously described approaches for anatomical muscle atrophy evaluation (31), with scores ranging from 0 (no abnormalities) to 4 (marked muscle hypotrophy). Pain sensitivity was not quantified using a standardized or validated pain scoring system, pressure algometry, or other objective measurement method. Therefore, it was considered a qualitative screening parameter used for baseline clinical characterization rather than a variable included in the statistical longitudinal analysis. The evaluations were documented through video recordings using the GoPro Hero9^®^ camera (California, United States) and performed by a blinded evaluator who actively participated in all reevaluations.

### 2.4 Thermographic evaluation of the axial skeleton

Horses were prepared and brushed 60 min before each thermographic assessment, kept stationary in their covered stall, and protected from solar radiation and airflow. Thermographic images were taken at 6:00 a.m (mean ambient temperature of 18.7 °C ± 3.8 °C). Images were captured using a FLIR ThermaCAM E25^®^ infrared camera (Teledyne, United States) with an emissivity of 0.98. The camera was positioned at 1.50 m or at a distance that allowed the observation and capture of images of the entire left and right lateral regions of the cervical spine, as well as a dorsal image of the thoracolumbar spine, highlighting the left and right sides. All thermographic images were acquired under standardized field conditions in each horse’s covered stall, protected from direct solar radiation and airflow. Assessments were performed in the early morning (approximately 6:00–7:00 a.m.) to reduce environmental variability. A standardized acquisition distance was maintained throughout the study, and the same evaluator performed all longitudinal image assessments. Ambient temperature was not actively controlled during image acquisition, which should be considered when interpreting thermographic comparisons. Additionally, the complete history of each horse was obtained, including any additional information that could directly impact the result of the images, such as night or day patrol exits, saddle marks, scars, wounds or abrasions, and recent treatments. Thermographic assessments were conducted at T0, T1, T2, T3, and T4 to observe any thermal pattern changes in the cervical and thoracolumbar regions before and after the treatment was administered to each group (32, 33). For anatomical coverage consistency, two dorsal thermographic images of the thoracolumbar region were obtained, while one left lateral and one right lateral image of the cervical region were acquired at each evaluation time point.

Thermographic assessments were interpreted using a semiquantitative evaluator-based scoring approach focused on symmetry and thermal heterogeneity patterns. Scores were defined as follows: score 0—homogeneous and symmetrical thermal pattern; score 1—mild asymmetry/heterogeneity; score 2—moderate asymmetry/heterogeneity; and score 3—marked asymmetry/heterogeneity. This approach was intended for comparative longitudinal assessment of thermal pattern changes rather than absolute thermographic quantification. No quantitative temperature extraction (e.g., maximum, minimum, or mean temperature values), emissivity-based thermal modeling, or formal calibration analysis was performed.

### 2.5 Mesotherapy

Following the ultrasonographic, thermographic, and clinical (time point T0), the animals were randomly subdivided into four groups. The cervical group was subdivided into Group 1A, consisting of horses treated with saline solution (SS) (*n* = 10), and Group 1B, consisting of horses treated with dexamethasone sulfate (DS) (0.05 mg/kg, Dexaflan^®^, Lema Injex Biologic, Brazil) (*n* = 10). The thoracolumbar spine group was subdivided into Group 2A, consisting of horses treated with SS (*n* = 10), and Group 2B, consisting of horses treated with DS (*n* = 10). The animals were randomly divided into each group (A or B) according to numerical classification and paper drawn in a brown envelope. The mesotherapy technique was administered by a single person who had no information about the selected treatment for each horse.

Mesotherapy was the only intervention applied to the horses throughout the entire study period. Immediately before the treatment, the target axial segment (cervical or thoracolumbar) was aseptically prepared using gauze soaked in 2% chlorhexidine scrubbing (Riodeine, Rioquímica S. A., Brazil) and then wiped with gauze soaked in 70% alcohol. A multi-injector with five sterile 4 mm needles (MesoDERM^®^, Alur Medical Ltda., Brazil) was used, attached to an intravenous extensor and a sterile 20 mL syringe.

The groups treated with SS received a total volume of 20 mL, while horses treated with DS were given a dose of 0.05 mg/kg, with a standard total volume of 10 mL (20 mg). Therefore, the syringe was filled with an additional 10 mL of saline solution, resulting in a total volume of 20 mL. The multi-injector and needles were positioned at a 30-degree angle to prevent them from penetrating beyond the dermal layer of the skin, ensuring that the substances remained at a superficial level. The application was performed on the cervical spine region, from C1 to C7, and on the thoracolumbar spine region, from T7 to L6, allowing for the formation of papules on both sides. Although the procedure is minimally invasive and only slightly painful, some animals required sedation to ensure the technique was performed properly. In this experiment, four of the 40 horses were sedated with detomidine (0.02 mg/kg, Detomidin^®^ 1%, Syntec, Brazil).

### 2.6 Statistical analysis

Score-based data were assessed for normality using four complementary tests: Kolmogorov–Smirnov, Anderson-Darling, Shapiro–Wilk, and D’Agostino-Pearson. All tests indicated non-normal distribution (*p* < 0.05), because the response variables were ordinal and repeatedly assessed over time, linear mixed models were used to account for longitudinal within-horse variation.

Linear mixed models (LMMs) were employed to analyze treatment effects, with individual horses included as random effects to account for repeated measures. Multiple comparisons were performed using Dunnett’s *post-hoc* test. All statistical analyses were conducted using GraphPad Prism software (version 8.3), with significance set at *p* < 0.05. Response variables were analyzed separately by anatomical region: three dependent variables for the cervical group (joint mobility, muscle hypotrophy, and thermographic patterns) and four for the thoracolumbar group (extension joint mobility, flexion joint mobility, muscle hypotrophy, and thermographic patterns).

## 3 Results

### 3.1 Baseline characteristics (T0)

#### 3.1.1 Ultrasonographic evaluation

At the ultrasonographic evaluation at T0, the cervical region showed a broader distribution of severity than the thoracolumbar groups. In the cervical group treated with saline solution (1A), mild changes predominated (70–7/10), whereas group 1B showed a higher proportion of moderate (40–4/10) and marked changes (30–3/10). In contrast, the thoracolumbar groups predominantly exhibited mild changes (60–90%) (Table 1). The anatomical distribution of changes was similar within each region, with C5–C6 as the most frequent cervical level (45–50% of cases) and T18–L1 accounting for most thoracolumbar changes (45–55%) (Tables 2, 3).

**Table 1:** Distribution of ultrasonographic lesion grades at baseline (T0) in the cervical (Groups 1A and 1B) and thoracolumbar (Groups 2A and 2B) regions.

Group	Mild	Moderate	Marked	Total (n)
Cervical 1A	7 (70%)	2 (20%)	1 (10%)	10
Cervical 1B	3 (30%)	4 (40%)	3 (30%)	10
Thoracolumbar 2A	9 (90%)	1 (10%)	0	10
Thoracolumbar 2B	6 (60%)	3 (30%)	1 (10%)	10

Data are presented as absolute numbers and percentages within each group. Ultrasonographic findings were classified as mild, moderate, or marked according to the predefined grading system. T0 = baseline.

**Table 2:** Distribution of ultrasonographic lesions across cervical joints at baseline (T0) in Groups 1A and 1B.

Group	C1-C2	C4-C5	C5-C6	C6-C7	Total (n)
Cervical 1A	1 (9%)	1 (9%)	5 (45.5%)	4 (36.1%)	11
Cervical 1B	0	3 (30%)	5 (50%)	2 (20%)	10

Data are presented as absolute numbers and percentages of affected joints within each group. The total number of affected joints may exceed the number of horses (*n* = 10 per group), as some horses presented abnormalities in more than one joint. T0 = baseline.

**Table 3:** Distribution of ultrasonographic lesions across thoracolumbar joints at baseline (T0) in Groups 2A and 2B.

Group	T12-T13	T13-T14	T15-T16	T16-T17	T18-L1	L1-L2	Total (n)
Thoracolumbar 2A	0	0	2 (18.8%)	2 (18.8%)	6 (54.5%)	1 (9%)	11
Thoracolumbar 2B	1 (6.6%)	1 (6.6%)	2 (13%)	3 (20%)	7 (46.6%)	1 (6.6%)	15

Data are presented as absolute numbers and percentages of affected joints within each group. The total number of affected joints may exceed the number of horses (*n* = 10 per group), as some horses presented abnormalities in more than one joint. T0 = baseline.

#### 3.1.2 Cervical spine groups

**Figure 3:**
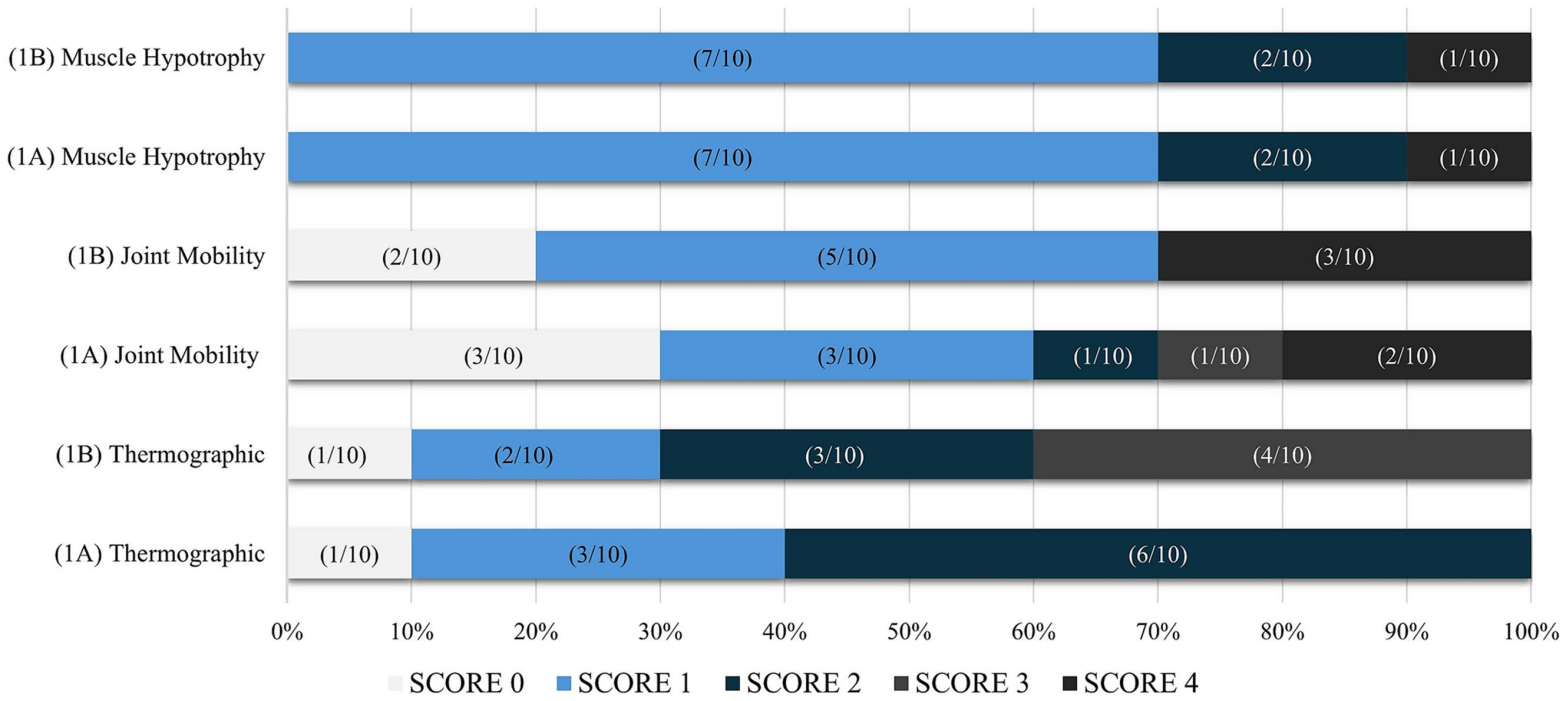
Baseline distribution of clinical and thermographic scores in the cervical region (Groups 1A and 1B). T0 represents the pre-treatment evaluation. Score 0: no alteration; Score 1: slight alteration; Score 2: mild alteration; Score 3: moderate alteration; and Score 4: marked alteration.

Clinical examination revealed regional differences in pain sensitivity between the treatment groups. In group 1A, 40% of the horses (4/10) exhibited pain sensitivity upon palpation of the atlantoaxial joint (C1–C2), compared to only 10% (1/10) in group 1B. Reduced joint mobility was identified in 70% (7/10) and 80% (8/10) of horses in groups 1A and 1B, respectively. All horses (100%) in both groups showed some degree of muscular hypotrophy, with varying levels of severity. Thermographic abnormalities were observed in 90% (9/10) of the horses in both cervical groups. However, the thermal pattern distribution differed significantly: group 1A predominantly exhibited cold spots (80%, 8/10) with minimal presence of hot spots (10%, 1/10), whereas group 1B showed a more balanced distribution between hot spots (40%, 4/10) and cold spots (50%, 5/10) (Figure 3).

#### 3.1.3 Thoracolumbar spine groups

**Figure 4:**
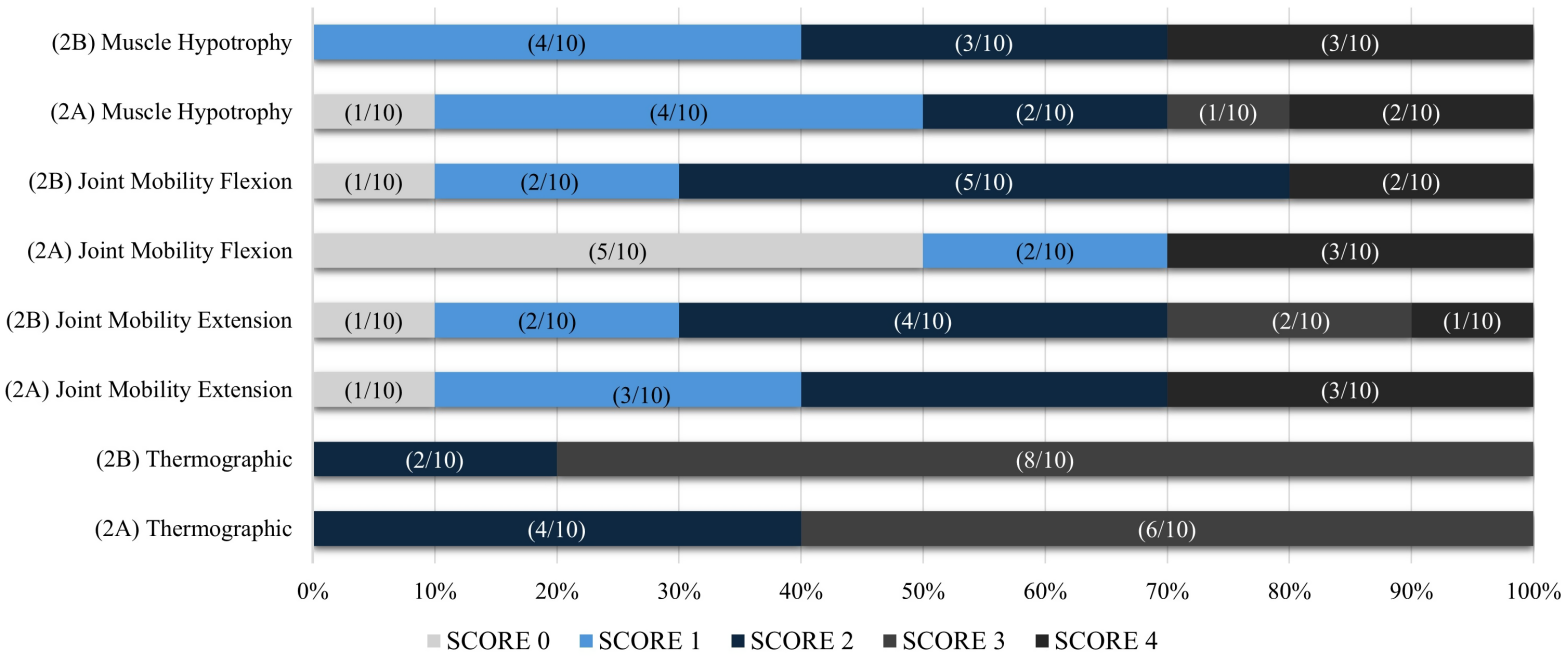
Baseline distribution of clinical and thermographic scores in the thoracolumbar region (Groups 2A and 2B). T0 represents the pre-treatment evaluation. Score 0: no alteration; Score 1: slight alteration; Score 2: mild alteration; Score 3: moderate alteration; and Score 4: marked alteration.

Pain sensitivity upon palpation differed substantially between thoracolumbar treatment groups, with 30% (3/10) of horses in the saline group (2A) and 80% (8/10) in the dexamethasone group (2B) demonstrating positive responses. Thermographic evaluation revealed abnormalities in both groups, with 100% (10/10) of horses displaying altered thermal patterns, characterized exclusively by hot spot predominance. Joint mobility in extension showed similar distribution between groups, with scores 2 and 3 being the most frequent. In contrast, flexion assessments revealed that 50% (5/10) of horses in group 2A had no alterations, while group 2B had 50% (5/10) of individuals showing mild reductions in mobility. Muscle hypotrophy was more pronounced in group 2B, with 30% of the horses scoring 2 and another 30% scoring 4. Group 2A exhibited a more heterogeneous distribution across scores 1–4 (Figure 4).

### 3.2 Treatment effects and temporal changes

#### 3.2.1 Cervical region (groups 1A and 1B)

**Figure 5:**
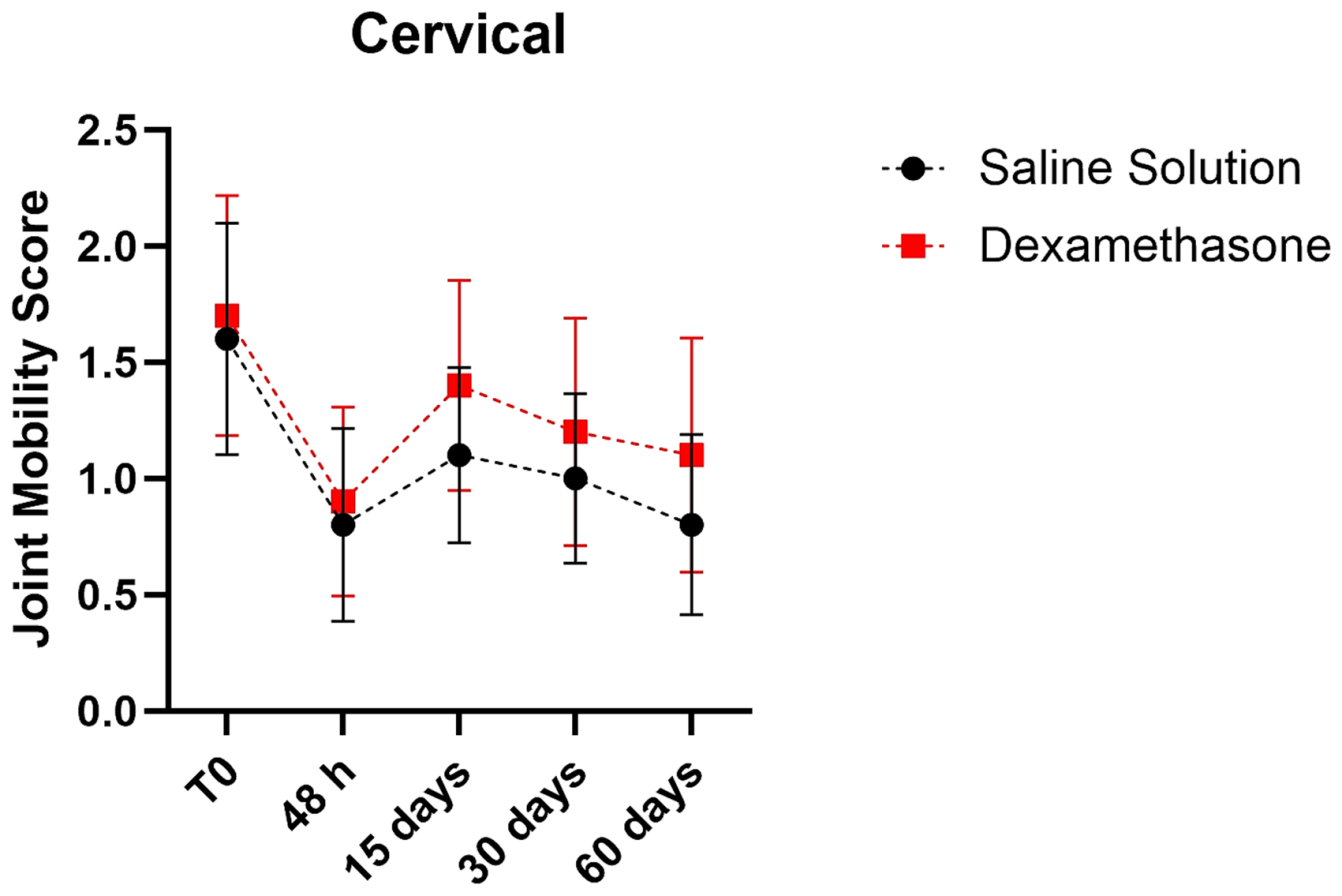
Temporal distribution of joint mobility scores in the cervical region of horses following mesotherapy. T0: baseline; T1: 48 h; T2: 15 days; T3: 30 days; and T4: 60 days post-treatment. Points and lines represent the mean joint mobility scores (± SEM) for each group (*n* = 10 animals per group). Group 1A: Saline solution; Group 1B: Dexamethasone (0.05 mg/kg). A significant temporal improvement in joint mobility was observed across both groups (*p* = 0.0309; Linear Mixed Model), with no statistically significant differences detected between the pharmacological agents (*p* > 0.05).

Linear mixed model analysis revealed a progressive improvement in cervical joint mobility over time [*F*(4, 72) = 2.85, *p* = 0.0309], in both groups (Figure 5). Neither treatment type (dexamethasone vs. saline) nor the time × treatment interaction reached statistical significance (*p* > 0.05), indicating that clinical improvement occurred similarly in both groups under the experimental conditions tested.

**Figure 6:**
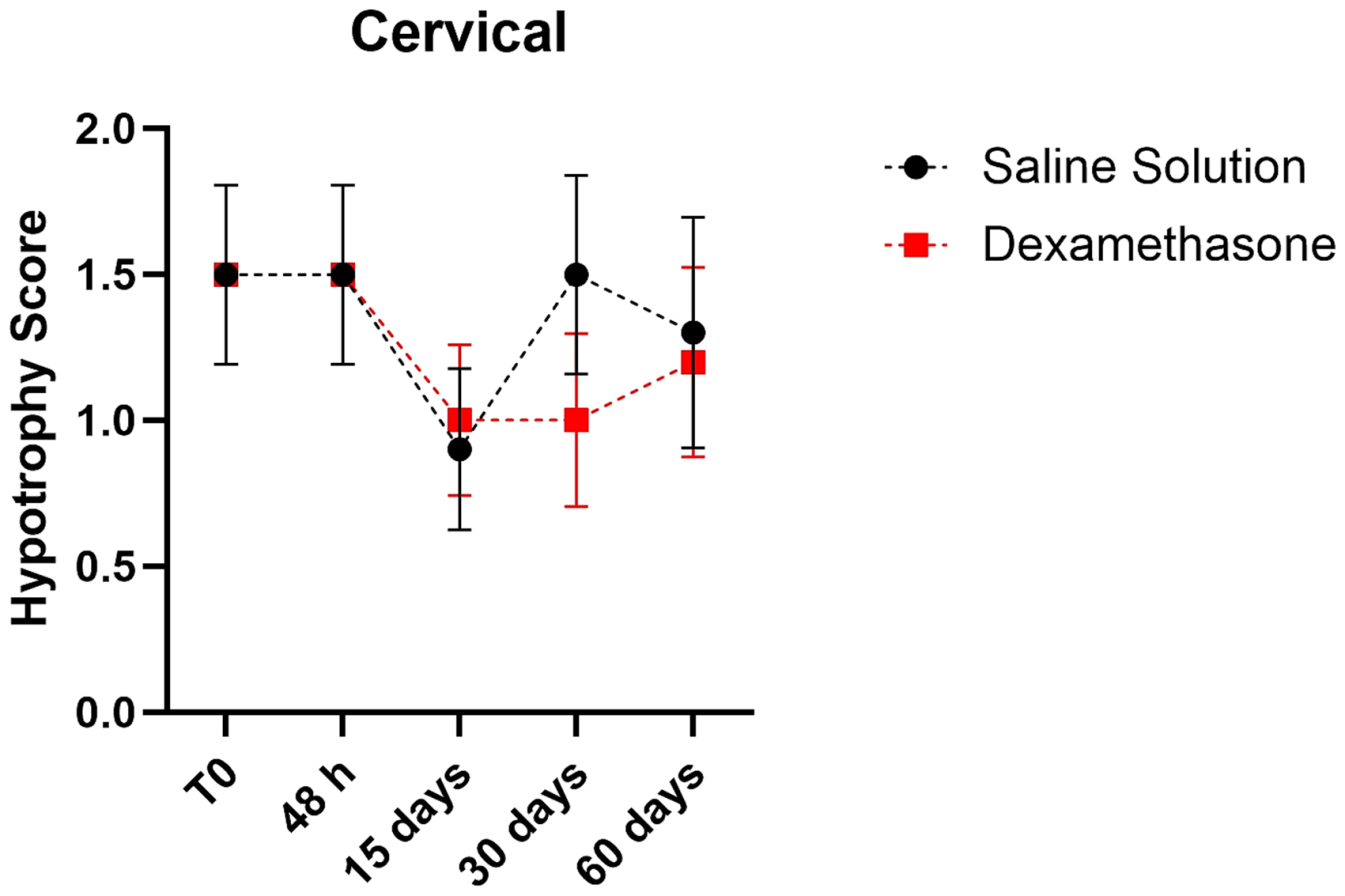
Temporal distribution of muscle hypotrophy scores in the cervical region of horses following mesotherapy. T0: baseline; T1: 48 h; T2: 15 days; T3: 30 days; and T4: 60 days post-treatment. Points and lines represent the mean muscle hypotrophy scores (± SEM) for each group (*n* = 10 animals per group). Group 1A: Saline solution; Group 1B: Dexamethasone (0.05 mg/kg). No statistically significant differences were detected between groups or across time points (*p* > 0.05; Linear Mixed Model).

No significant effects were detected for treatment, time, or their interaction on cervical muscle hypotrophy scores (all *p* > 0.05) (Figure 6), suggesting that the 60-day follow-up period was insufficient to detect measurable changes in muscle mass parameters.

**Figure 7:**
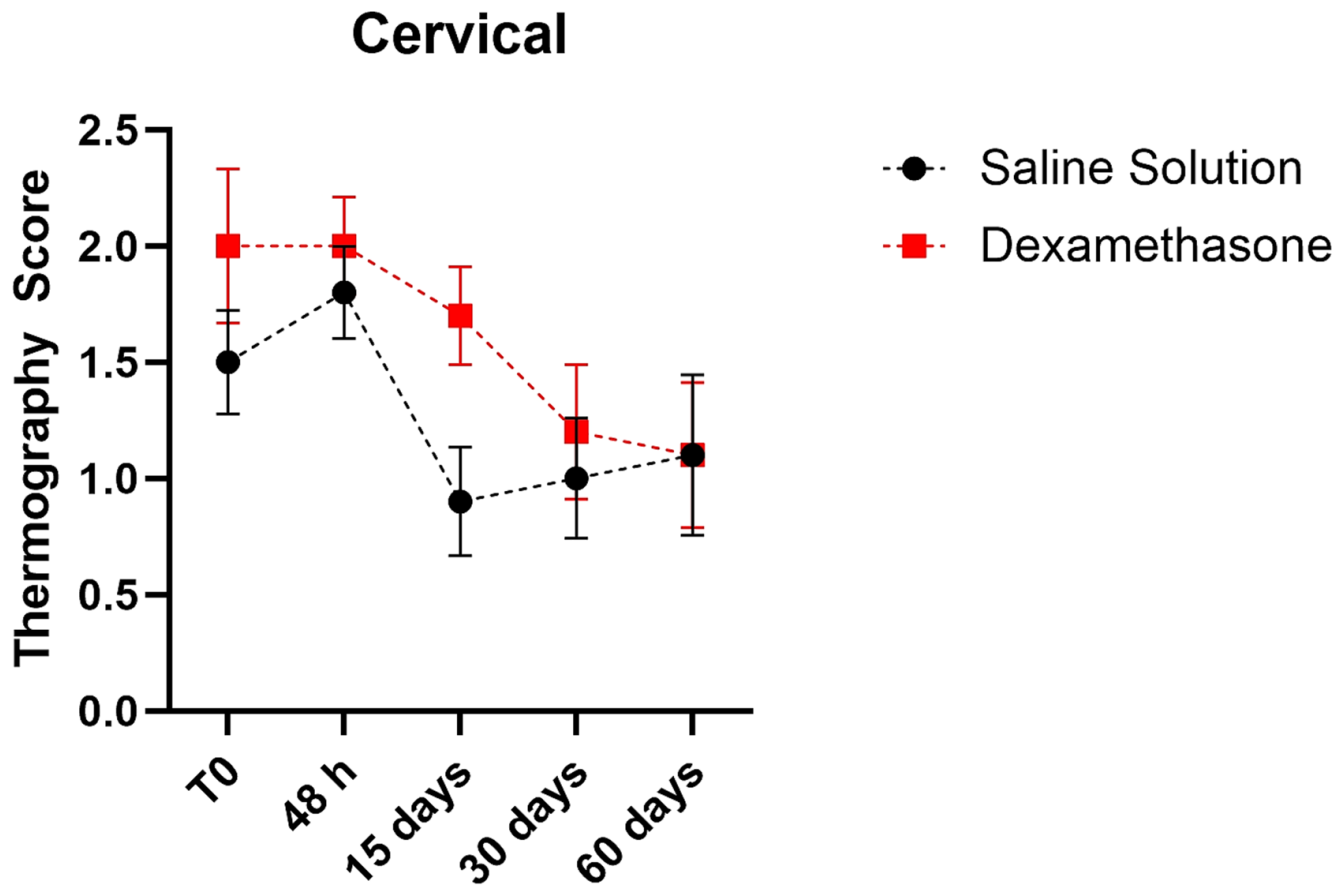
Temporal distribution of thermographic scores in the cervical region of horses following mesotherapy. T0: baseline; T1: 48 h; T2: 15 days; T3: 30 days; and T4: 60 days post-treatment. Points and lines represent the mean thermographic scores (± SEM) for each group (*n* = 10 animals per group; analysis conducted per joint). Group 1A: Saline solution; Group 1B: Dexamethasone (0.05 mg/kg). Asterisks indicate significant reduction compared to baseline (T0) within each group: (*) *p* < 0.05; (**) *p* < 0.01 (Linear Mixed Model followed by Dunnett’s *post-hoc* test).

In contrast, thermographic assessment appeared comparatively more responsive for identifying temporal pattern changes in this region under the present semiquantitative evaluation conditions, showing significant effects for both time [*F*(3.047, 68.55) = 3.893, *p* = 0.0121] and treatment [*F*(1, 90) = 4.033, *p* = 0.0476] (Figure 7). These findings suggest that thermographic assessment appeared comparatively more responsive than the evaluated clinical variables for detecting temporal pattern changes under the semiquantitative study conditions.

#### 3.2.2 Thoracolumbar region (groups 2A and 2B)

Analysis of thoracolumbar variables revealed a consistent pattern in which time emerged as the sole significant factor across all measured parameters (*p* < 0.05), whereas treatment type and time × treatment interaction remained non-significant (*p* > 0.05).

**Figure 8:**
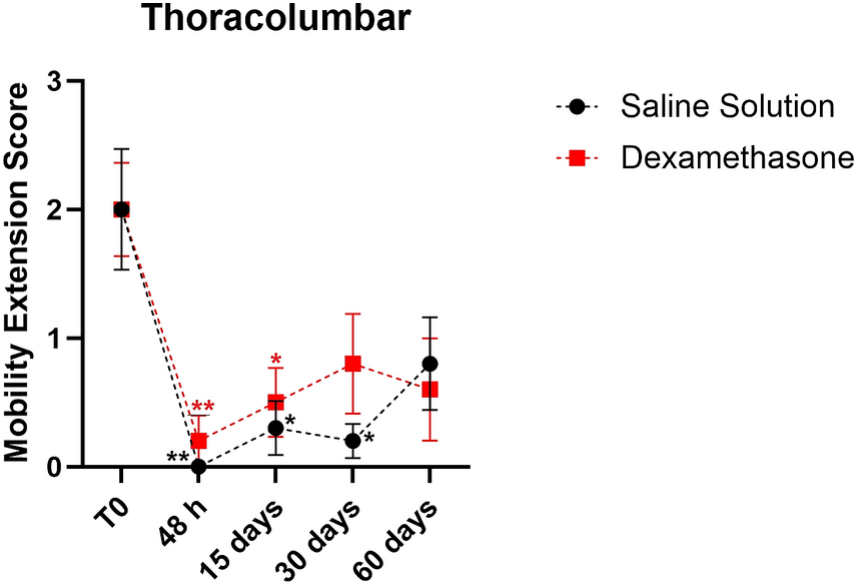
Temporal distribution of joint mobility scores in extension for the thoracolumbar region of horses following mesotherapy. T0: baseline; T1: 48 h; T2: 15 days; T3: 30 days; and T4: 60 days post-treatment. Points and lines represent the mean joint mobility scores in extension (± SEM) for each group (*n* = 10 animals per group; analysis conducted per joint). Group 2A: Saline solution; Group 2B: Dexamethasone (0.05 mg/kg). Asterisks indicate significant reduction compared to baseline (T0) within each group: (*) *p* < 0.05; (**) *p* < 0.01 (Linear Mixed Model followed by Dunnett’s *post-hoc* test). Significant improvement was sustained until T3 in Group 2A and T2 in Group 2B.

**Figure 9:**
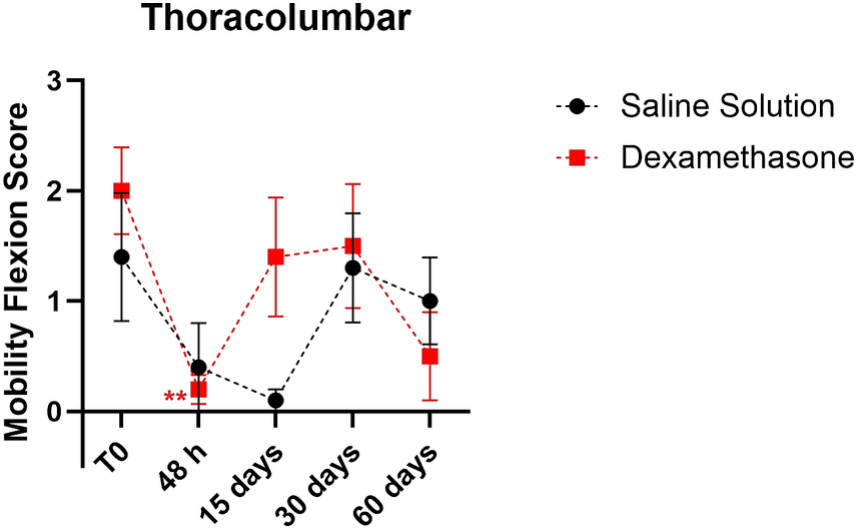
Temporal distribution of joint mobility scores in flexion for the thoracolumbar region of horses following mesotherapy. T0: baseline; T1: 48 h; T2: 15 days; T3: 30 days; and T4: 60 days post-treatment. Points and lines represent the mean joint mobility scores in flexion (± SEM) for each group (*n* = 10 animals per group; analysis conducted per joint). Group 2A: Saline solution; Group 2B: Dexamethasone (0.05 mg/kg). A double asterisk indicates a significant reduction compared to baseline (T0) at 48 h in Group 2B: (**) *p* < 0.01 (Linear Mixed Model followed by Dunnett’s *post-hoc* test).

Both extension and flexion mobility demonstrated significant temporal effects, with *F*(2.257, 40.63) = 14.17, *p* < 0.0001 for extension and *F*(3.250, 58.50) = 4.322, *p* = 0.0067 for flexion (Figures 8, 9). *Post-hoc* analysis revealed distinct response patterns: extension mobility in the saline group (2A) showed significant improvement until T3 (30 days), whereas the dexamethasone group (2B) maintained improvement only until T2 (15 days) (Figure 8). In contrast, flexion mobility improvement was limited to T1 (48 h) in group 2B and no significant changes detected in group 2A (Figure 9).

**Figure 10:**
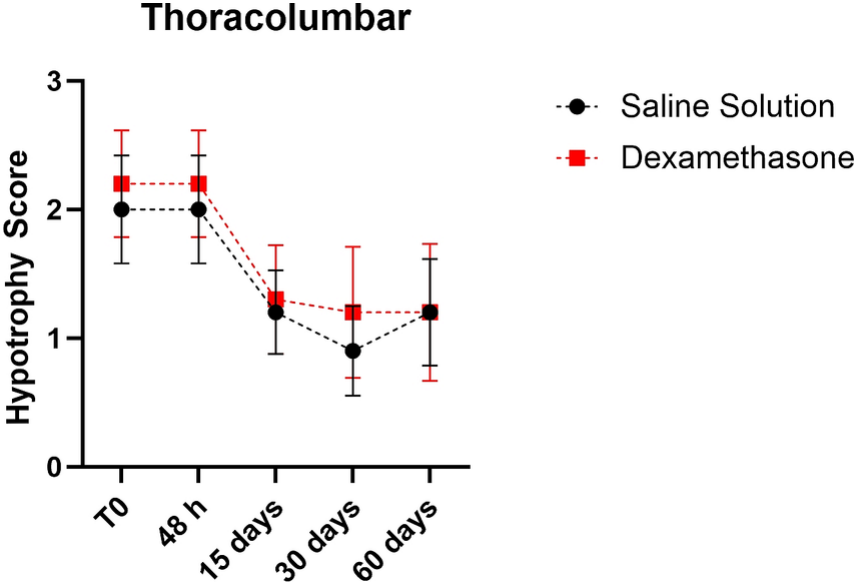
Temporal distribution of muscle hypotrophy scores in the thoracolumbar region of horses following mesotherapy. T0: baseline; T1: 48 h; T2: 15 days; T3: 30 days; and T4: 60 days post-treatment. Points and lines represent the mean muscle hypotrophy scores (± SEM) for each group (*n* = 10 animals per group). Group 2A: Saline solution; Group 2B: Dexamethasone (0.05 mg/kg). A highly significant temporal effect was observed (*p* < 0.0001; Linear Mixed Model), although *post-hoc* comparisons did not identify specific time points that differed significantly from baseline in either group.

Thoracolumbar muscle hypotrophy also showed a highly significant time effect [*F*(1.922, 34.60) = 22.33, *p* < 0.0001], although *post-hoc* comparisons did not identify specific time points that differed significantly from baseline in either group (Figure 10). This pattern suggests a gradual and continuous improvement in muscle condition rather than a discrete change at isolated time points.

**Figure 11:**
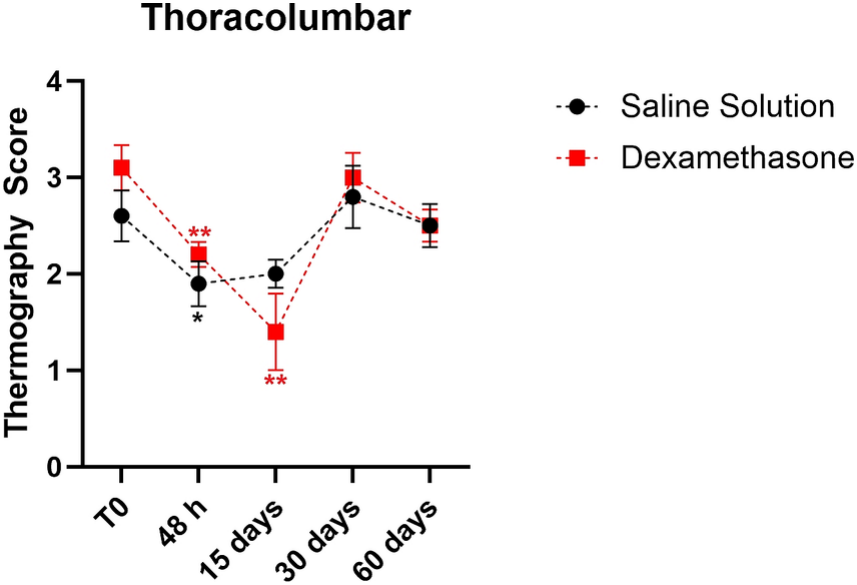
Temporal distribution of thermographic scores in the thoracolumbar region of horses following mesotherapy. T0: baseline; T1: 48 h; T2: 15 days; T3: 30 days; and T4: 60 days post-treatment. Points and lines represent the mean thermographic scores (± SEM) for each group (*n* = 10 animals per group; analysis conducted per joint). Group 2A: Saline solution; Group 2B: Dexamethasone (0.05 mg/kg). Asterisks indicate significant reduction compared to baseline (T0) within each group: (*) *p* < 0.05; (**) *p* < 0.01 (Linear Mixed Model followed by Dunnett’s *post-hoc* test). Significant thermal improvement was sustained until T1 in Group 2A and until T2 in Group 2B.

Thermographic pattern analysis likewise demonstrated significant temporal changes [*F*(2.848, 51.26) = 13.74, *p* < 0.0001] (Figure 11). Saline-treated horses (2A) showed significant thermal improvement until T1 (48 h), whereas dexamethasone-treated horses (2B) showed improvement until T2 (15 days).

## 4 Discussion

The present study demonstrated temporal changes in selected clinical and thermographic parameters following both saline- and dexamethasone-based mesotherapy protocols in horses with cervical and thoracolumbar spinal disorders. Improvements over time were primarily observed in joint mobility and thermographic patterns, particularly in the thoracolumbar region, whereas muscle hypotrophy showed more limited and descriptive longitudinal changes. Importantly, no statistically significant differences were identified between treatment protocols for most evaluated outcomes under the tested experimental conditions. These findings suggest that both active mesotherapy approaches were associated with comparable responses over time, while thermographic assessment appeared comparatively more responsive for detecting longitudinal pattern changes under the semiquantitative study conditions.

Clinical evaluation through systematic inspection and palpation of the vertebral column is an essential tool for identifying musculoskeletal abnormalities in horses, particularly regarding muscle hypotrophy, stiffness, pain, and postural asymmetries. These findings are common in horses with cervical, thoracolumbar, and lumbosacral pain and are associated with vertebral instability and secondary muscular compensations that reduce mobility and alter biomechanics (1, 10, 34). Previous studies support these observations such as Melo and Ferreira (35) reported hypotrophy of the multifidus dorsi muscle in 86.6% of horses diagnosed with back pain. In the present study, at baseline (T0), 85% (17/20) of the horses exhibited some degree of reduced mobility during thoracolumbar extension, and 65% (13/20) during flexion, and 75% (15/20) showed cervical movement restriction, confirming a high prevalence of axial dysfunction in the study population.

The observed coexistence of reduced mobility and muscle hypotrophy supports the hypothesis that muscular dysfunction may be closely associated with impaired axial biomechanics, as also suggested by Findley and Singer (30), who emphasized the importance of deep muscular stability in maintaining axial function in athletic horses. Muscle hypotrophy in horses with axial dysfunction may reflect disuse, altered biomechanics, chronic compensatory stabilization, and reduced activation of deep epaxial musculature. In our study, in addition to the high percentage of animals presenting joint mobility restrictions, a high prevalence of muscle hypotrophy was also observed in both the cervical and thoracolumbar regions. Additional factors such as advanced age (mean of 15 years), intensive use for patrol and equine-assisted therapy, rider weight, and potential lameness may also have acted as predisposing or aggravating contributors (7, 8).

Regarding joint mobility, temporal improvement was observed in both anatomical regions, particularly in the thoracolumbar groups (2A and 2B), with the most notable progress seen in extension mobility. The saline-treated group (2A) maintained significant improvement until day 30 (T3), whereas the dexamethasone-treated group (2B) maintained it only until day 15 (T2). In the cervical groups (1A and 1B), improvements were more limited and primarily observed during early follow-up, suggesting a shorter-term clinical response under the tested conditions. However, no statistically significant differences were identified between treatment protocols. Repeated treatment approaches have been explored in other musculoskeletal contexts, including knee osteoarthritis (36, 37) and chronic low back pain (21); however, the optimal treatment frequency or duration for mesotherapy in equine spinal disorders remains undefined and warrants further investigation.

Thermographic evaluation appeared comparatively more responsive in detecting longitudinal thermal pattern changes, particularly during early post-treatment assessments. Injured tissues may present altered circulation patterns that can be reflected in thermographic asymmetry or heterogeneity, including focal hot spots associated with increased local perfusion and inflammatory activity, or cold regions related to altered vascular or autonomic responses (32, 38). In the thoracolumbar region, thermal improvements persisted until T1 (48 h) in the saline-treated group (2A) and until T2 (15 days) in the dexamethasone-treated group (2B), suggesting a descriptively prolonged response in the latter, although no statistically significant between-treatment differences were identified. In the cervical region, thermographic changes were mainly evident at T2 and T3, with significant effects observed for both time and treatment. These observations support the potential utility of thermography as a complementary longitudinal assessment tool for monitoring regional physiological pattern changes, consistent with previous reports involving equine muscular imbalance and spinal dysfunction (39, 40). However, because thermographic interpretation in this study was semiquantitative and pattern-based, direct physiological or mechanistic attribution should be interpreted cautiously.

Regarding muscle hypotrophy, no statistically significant effects of treatment type or treatment × time interaction were detected in either anatomical region. In the cervical groups, no significant temporal changes were identified, whereas the thoracolumbar region showed a significant time effect, although *post-hoc* analyses did not reveal discrete time points that differed significantly from baseline. This pattern may suggest gradual longitudinal changes rather than abrupt structural improvement. The follow-up period may have been insufficient to detect more pronounced muscular adaptations, particularly in the absence of a targeted rehabilitation or physical training protocol. Supporting this interpretation, Stubbs et al. (41) demonstrated that ultrasonographic changes consistent with hypertrophy of the multifidus dorsi muscle were identified only after prolonged targeted exercise. Although no robust statistical changes were observed, descriptive reductions in muscle hypotrophy scores were noted in both anatomical regions, particularly during longitudinal follow-up in the thoracolumbar groups. These observations may reflect functional recovery patterns or gradual muscular adaptation that were not fully captured by semiquantitative clinical scoring alone. Similar delayed morphological recovery has also been described after mesotherapy-based rehabilitation in other species, including dogs with lumbar dysfunction, where progressive muscular improvement was associated with restoration of functional use (26). In this context, continuous follow-up using more objective tools, such as serial ultrasonography or quantitative muscle assessment, may be important for future studies, particularly considering the functional role of epaxial musculature in axial stability and locomotor biomechanics, as highlighted in previous studies involving equine spinal dysfunction and multifidus-related muscular compromise (13, 35).

Although both mesotherapy protocols were associated with temporal clinical and thermographic changes, the biological mechanisms underlying these responses cannot be directly established from the present study. Localized procedural factors such as intradermal stimulation, microneedling, local neurovascular modulation, and transient inflammatory responses may potentially contribute to the observed findings, as suggested in previous mesotherapy-related investigations (16, 19, 42, 43). Similarly, Ferrara et al. (44) reported comparable pain relief in human chronic spinal conditions treated with saline and anti-inflammatory mesotherapy protocols, suggesting that localized procedural stimulation may contribute to part of the therapeutic response. However, because both saline- and dexamethasone-based protocols demonstrated comparable responses across most evaluated outcomes, the relative contributions of pharmacological and procedural components remain uncertain and should be explored in future controlled investigations.

Although temporal improvements were observed in joint mobility and thermographic parameters following both mesotherapy protocols, these findings should be interpreted within the constraints of the study design. The present study was structured as a randomized comparative trial between two active intradermal treatment protocols (saline solution and dexamethasone), rather than as a placebo-controlled, sham-controlled, or equivalence trial. Consequently, the absence of statistically significant differences between treatments should not be interpreted as evidence of therapeutic equivalence, superiority, or true absence of biological differences between interventions. Instead, the findings indicate that both active protocols were associated with comparable responses under the tested experimental conditions. Future sham-controlled or untreated comparative studies will be important to better define the relative therapeutic and procedural contributions of mesotherapy in equine spinal disorders.

Another important limitation relates to the semiquantitative and partially subjective nature of the outcome measures used in this study. Joint mobility, muscle hypotrophy, and thermographic assessments were based on investigator-developed structured ordinal scoring systems created for longitudinal comparative evaluation and informed by previously described clinical and imaging interpretation methods. However, these scores were not directly derived from validated numerical scales and were not formally validated for this specific application. Although the use of a single blinded experienced evaluator improved consistency during repeated assessments, formal interobserver reliability, intraobserver repeatability, and calibration analyses were not performed. Therefore, these findings should be interpreted considering potential subjectivity, reproducibility limitations, and reduced measurement precision.

Additional caution is required when interpreting thermographic findings. The thermographic analysis was based on semiquantitative visual pattern interpretation rather than absolute thermal extraction, and environmental temperature was not actively controlled during image acquisition. Although thermographic conditions were standardized as much as possible under field conditions, thermography remains sensitive to external, environmental, and physiological confounding factors. Therefore, thermographic observations should be interpreted primarily as comparative longitudinal pattern responses rather than precise quantitative thermal measurements or definitive indicators of localized pathological activity.

An additional limitation relates to clinical characterization of the study population. Although horses presented ultrasonographic spinal abnormalities and clinical indicators potentially compatible with axial dysfunction, signs such as lameness, locomotor changes, and discomfort during saddling were not specific to spinal pathology and may reflect multifactorial conditions. Therefore, the enrolled horses should not be interpreted exclusively as confirmed clinical back pain cases, which should be considered when extrapolating broader clinical applicability.

A final statistical limitation relates to sample size. As no *a priori* sample size or formal power calculation was performed and the study population was based on a convenience sample of eligible horses, the relatively small group sizes may have limited statistical power to detect subtle between-group differences. Consequently, the possibility of Type II error cannot be excluded, and the absence of detectable differences should not be interpreted as evidence of therapeutic equivalence or true absence of biological differences between treatment protocols. Collectively, these limitations indicate that the present findings should be interpreted primarily as exploratory comparative observations that may support future controlled investigations into the clinical and mechanistic role of mesotherapy in equine spinal disorders.

## 5 Conclusion

Mesotherapy remains a minimally invasive technique with potential applicability in equine rehabilitation and management of musculoskeletal dysfunctions. In this study, both dexamethasone and saline-based mesotherapy protocols were associated with temporal improvements in selected clinical and thermographic parameters in horses with cervical and thoracolumbar spinal abnormalities. However, no statistically significant differences were detected between treatment protocols under the tested experimental conditions, and these findings should be interpreted considering the exploratory design and limited sample size.

Future studies including larger sample sizes, objective outcome measures, sham-treated or untreated control groups, and extended follow-up periods are warranted to better define the therapeutic role and mechanistic contributions of mesotherapy in equine spinal disorders.

## Data Availability

The original contributions presented in the study are included in the article/supplementary material, further inquiries can be directed to the corresponding author.
